# Emergency colorectal cancer in old and very elderly patients: a narrative review

**DOI:** 10.3389/fonc.2026.1816546

**Published:** 2026-05-15

**Authors:** Carlo Bergamini, Jacopo Martellucci, Davina Perini, Paolo Prosperi, Alessio Giordano

**Affiliations:** Department of Emergency and Acceptance, Emergency Surgery Unit, Careggi University Hospital, Florence, Italy

**Keywords:** colorecal cancer, elderly, emergency, obstruction, octuagenarian

## Abstract

**Introduction:**

The progressive aging of the global population has led to a substantial increase in colorectal cancer (CRC) incidence among very elderly individuals. Patients aged ≥80 years represent a highly vulnerable subgroup characterized by frailty, multimorbidity, reduced physiological reserve, and a high likelihood of emergency presentation. Management of CRC emergencies in this population requires individualized decision-making that balances oncologic radicality with functional preservation and patient-centered goals.

**Methods:**

A structured narrative review was conducted using PubMed, Embase, and Scopus. Studies addressing emergency presentation and management of CRC in elderly and very elderly patients were analyzed, with specific focus on populations aged ≥80 years. Evidence was synthesized into pragmatic clinical frameworks integrating oncologic and geriatric principles.

**Results:**

Emergency CRC presentation occurs in up to 46% of colon cancers among patients older than 80 years and is consistently associated with worse short- and long-term survival. Malignant bowel obstruction is the most frequent emergency scenario. Bridge-to-surgery strategies, including self-expanding metal stents (SEMS) or diverting stomas, significantly reduce early mortality compared with emergency resection in selected elderly patients. Perforation and septic complications require damage-control approaches prioritizing rapid source control and physiological stabilization. Frailty and comorbidity burden are major independent prognostic determinants across all emergency presentations.

**Conclusions:**

Emergency CRC in patients aged ≥80 years represents a high-risk clinical scenario requiring integration of oncologic rigor with geriatric-oriented care. Future research should prioritize geriatric-specific endpoints, predictive frailty-based triage models, and real-world functional outcomes. These findings highlight the urgent need for geriatric-tailored emergency surgical pathways.

## Highlights

- Emergency CRC surgery in patients ≥80 years is high risk, driven primarily by frailty and comorbidities.
- Decision-making should be goal-oriented and patient-centered.
- Bridge-to-surgery strategies reduce early mortality in selected elderly patients.
- Damage-control surgery should prevail in unstable patients.
- Future studies should develop frailty-based triage models and geriatric-focused care pathways.

## Introduction

Population aging is reshaping the epidemiology of colorectal cancer (CRC). Individuals aged ≥80 years represent one of the fastest-growing oncologic populations worldwide, with a parallel rise in emergency presentations including obstruction, perforation, hemorrhage, and sepsis.

Emergency CRC presentation follows a marked age-related gradient and is consistently associated with poorer oncologic and functional outcomes compared with elective diagnosis (1, 2). Elderly patients more frequently present with advanced tumor stage, incomplete staging, and limited eligibility for multimodal therapy (3, 4). Advanced age, frailty, and comorbidity burden significantly influence postoperative outcomes and treatment allocation in elderly colorectal cancer patients (5–7).

In this context, management extends beyond tumor resectability. Age-related physiological decline, frailty, polypharmacy, and multimorbidity substantially reduce tolerance to surgical stress and impair postoperative recovery. Decision-making must therefore integrate oncologic principles with comprehensive geriatric assessment and clearly defined patient-centered goals (8).

This review provides a pragmatic, evidence-based overview of CRC emergencies in patients aged ≥80 years, integrating surgical strategy with geriatric-oriented clinical reasoning.

## Methods

A structured narrative review was conducted using PubMed, Embase, and Scopus to identify literature on emergency presentation and management of CRC in elderly and very elderly patients, focusing on individuals aged ≥80 years. Controlled vocabulary and free-text terms related to CRC, emergency surgery, frailty, and geriatric outcomes were applied.

Priority was given to population-based registries, international guidelines, multicenter audits, systematic reviews, and clinically relevant observational studies published in English between 2000 and 2025. Although no formal risk-of-bias assessment was performed, methodological quality and proportionality between oncologic benefit and geriatric risk were critically appraised. To reduce selection bias, emphasis was placed on high-quality evidence including systematic reviews, international guidelines, and large population-based studies. When evidence was limited, this was explicitly acknowledged.

## Results

### Malignant large bowel obstruction

**Figure 1:**
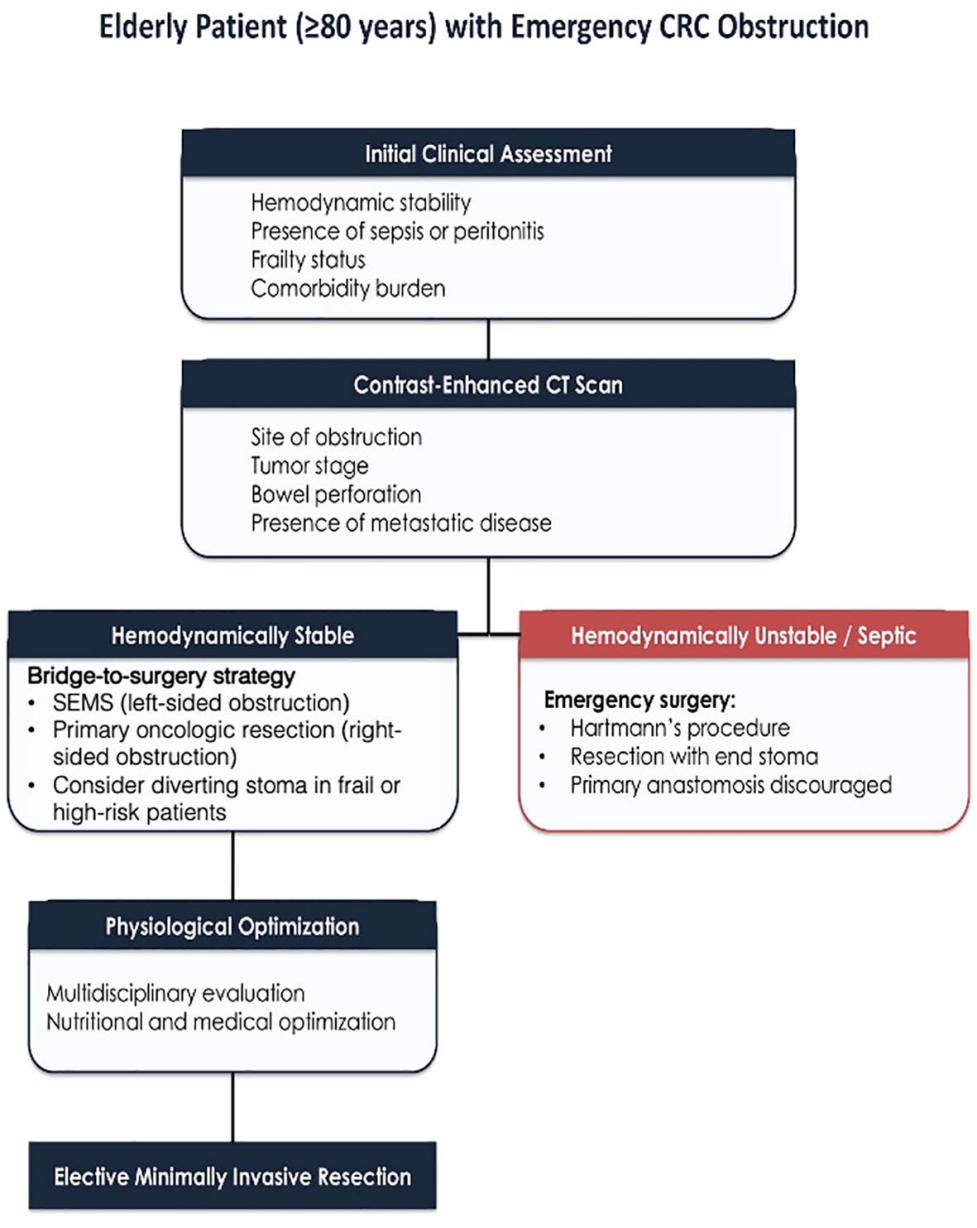
Proposed management algorithm for obstructive colorectal cancer in patients aged ≥80 years. The flowchart highlights the role of hemodynamic stability, frailty-based stratification, and goal-of-care decision-making in guiding treatment selection between bridge-to-surgery strategies and emergency surgical intervention.

Malignant bowel obstruction is the most frequent CRC emergency in elderly patients and often requires urgent decompression to prevent ischemia or perforation. Delayed diagnosis due to nonspecific symptoms frequently results in advanced-stage disease at presentation. Management requires assessment of hemodynamic stability, tumor location, stage, and functional status (9, 10) (**Figure 1**).

### Left-sided colon obstruction

The management of left-sided obstructing colon cancer (LSOCC) has evolved significantly with the adoption of bridge-to-surgery strategies. Emergency resection, historically standard, is associated with high morbidity and mortality in elderly patients.

Dutch ColoRectal Audit data involving over 4,500 patients demonstrated 30-day mortality exceeding 6% after emergency resection versus approximately 1% following decompressive staged surgery (11). In patients older than 70 years, mortality approached 9.5% after emergency surgery compared with 1.6% after bridge strategies (12).

Bridge-to-surgery options include SEMS placement and diverting stoma creation, allowing physiological optimization, nutritional recovery, multidisciplinary evaluation, and increased feasibility of minimally invasive resection. Reduced permanent stoma rates have also been reported (13, 14). National guideline implementation studies confirm improved outcomes and reduced emergency resections following systematic adoption of decompressive strategies (15, 16). Recent evidence suggests that frailty-based selection rather than chronological age alone is the primary determinant of benefit from bridge-to-surgery strategies.

### Right-sided colon obstruction

Obstructing right-sided colon cancer (RSCC) differs clinically from left-sided obstruction. The wider lumen and liquid fecal content often produce a more insidious presentation characterized by anemia and subacute obstruction. When acute occlusion occurs, patients frequently present with advanced disease and physiological deterioration.

Unlike left-sided disease, bridge-to-surgery strategies remain controversial in RSCC. Although technically feasible, right-sided stenting is less commonly performed due to anatomical variability and limited evidence regarding long-term oncologic safety. Current guidelines generally recommend primary oncologic resection in hemodynamically stable patients, as right hemicolectomy can often be performed without staged procedures or protective stomas (17).

In elderly individuals, emergency right hemicolectomy must be carefully balanced against frailty and comorbidity burden. While primary anastomosis is traditionally considered safer than in left-sided surgery, postoperative morbidity remains substantial in octogenarians, particularly in the presence of malnutrition, anemia, or sepsis. In unstable or severely frail patients, damage-control strategies with delayed anastomosis or temporary diversion may be safer.

When feasible and performed by experienced teams in stable patients, minimally invasive surgery may reduce pulmonary complications and facilitate recovery.

Management of RSCC in patients aged ≥80 years should prioritize physiological stabilization and individualized proportionality, integrating oncologic radicality with geriatric assessment principles. Accordingly, in stable patients, primary oncologic resection should be considered the preferred approach, while diverting stoma may be reserved for frail or high-risk individuals.

### Obstructing rectal cancer

Evidence supporting decompressive strategies in obstructing rectal cancer is more limited. Technical constraints, pelvic anatomy, and concerns regarding oncologic planes restrict widespread stent use. However, multidisciplinary consensus suggests selective stent placement in high rectal obstruction when technically feasible and performed by experienced endoscopists (18, 19).

### Palliative obstruction

In metastatic disease or limited life expectancy, treatment goals shift toward symptom control and quality of life preservation. SEMS placement has been associated with shorter hospitalization and higher rates of home discharge compared with diverting stomas (18, 19). Systematic reviews report substantial variability in postoperative outcomes, with median survival often below one year in carcinomatosis-related obstruction (20). Careful patient selection and goal-concordant care remain essential.

### Perforation, peritonitis, and sepsis

**Figure 2:**
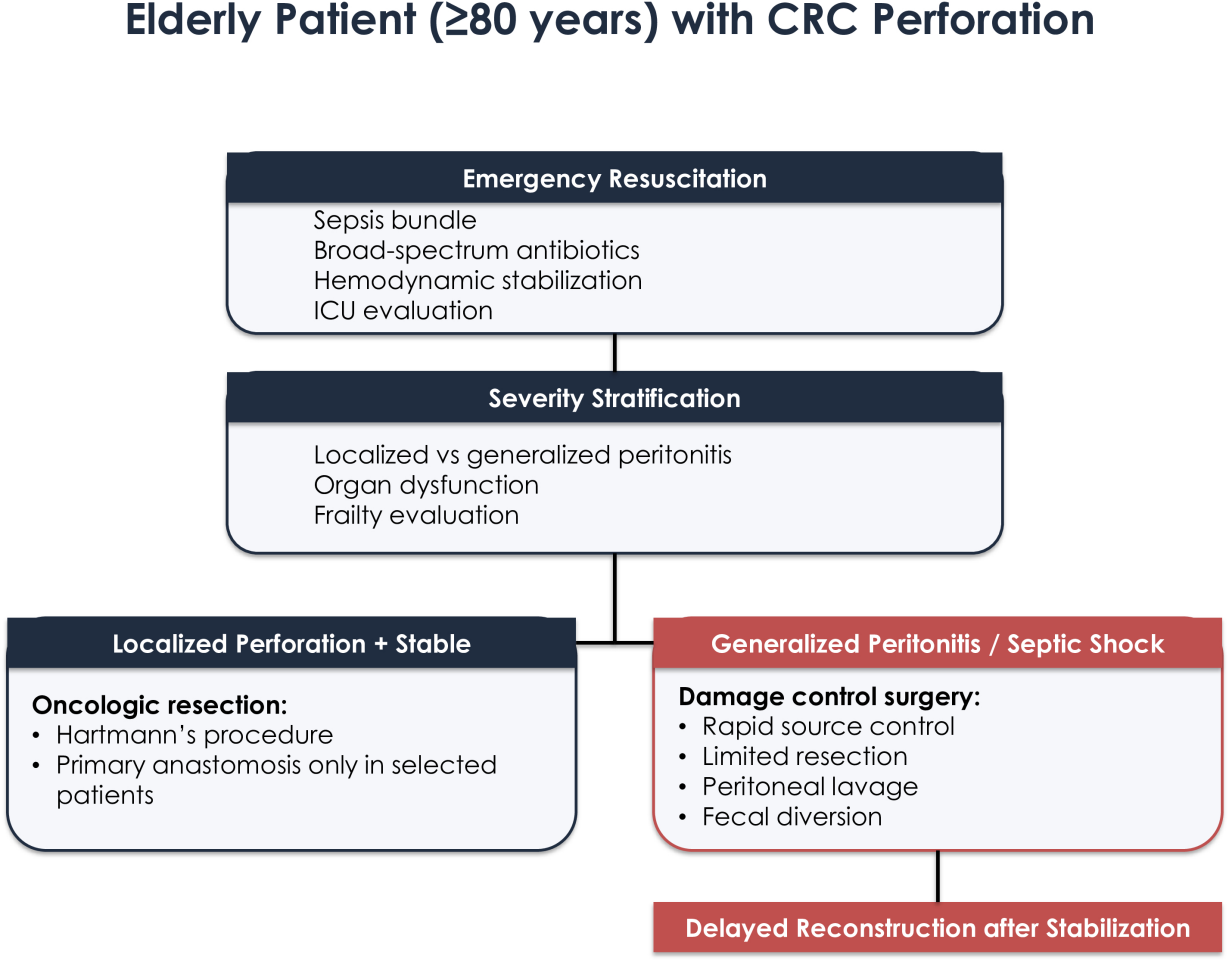
Decision-making pathway for perforated colorectal cancer presenting with peritonitis or sepsis in elderly patients. The algorithm emphasizes the role of damage-control surgery in unstable patients and staged reconstruction following physiological recovery.

Perforated CRC represents the most life-threatening emergency presentation. Mortality ranges from 20% to over 40% in frail octogenarians (21, 22) (**Figure 2**).

Septic physiology is often amplified in elderly patients due to immunosenescence and delayed diagnosis (23, 24). Guidelines recommend staged procedures in unstable patients, with Hartmann’s procedure remaining the most accepted strategy for effective source control (16).

Damage-control surgery—rapid resection, lavage, and diversion with delayed reconstruction—reduces physiological stress and improves stabilization (25, 26). Postoperative management including early goal-directed therapy, infection control, and multidisciplinary ICU support is critical. In elderly septic patients, outcomes are strongly influenced by early geriatric co-management and ICU-level support, which remain underrepresented in current surgical literature.

### Acute hemorrhage

**Figure 3:**
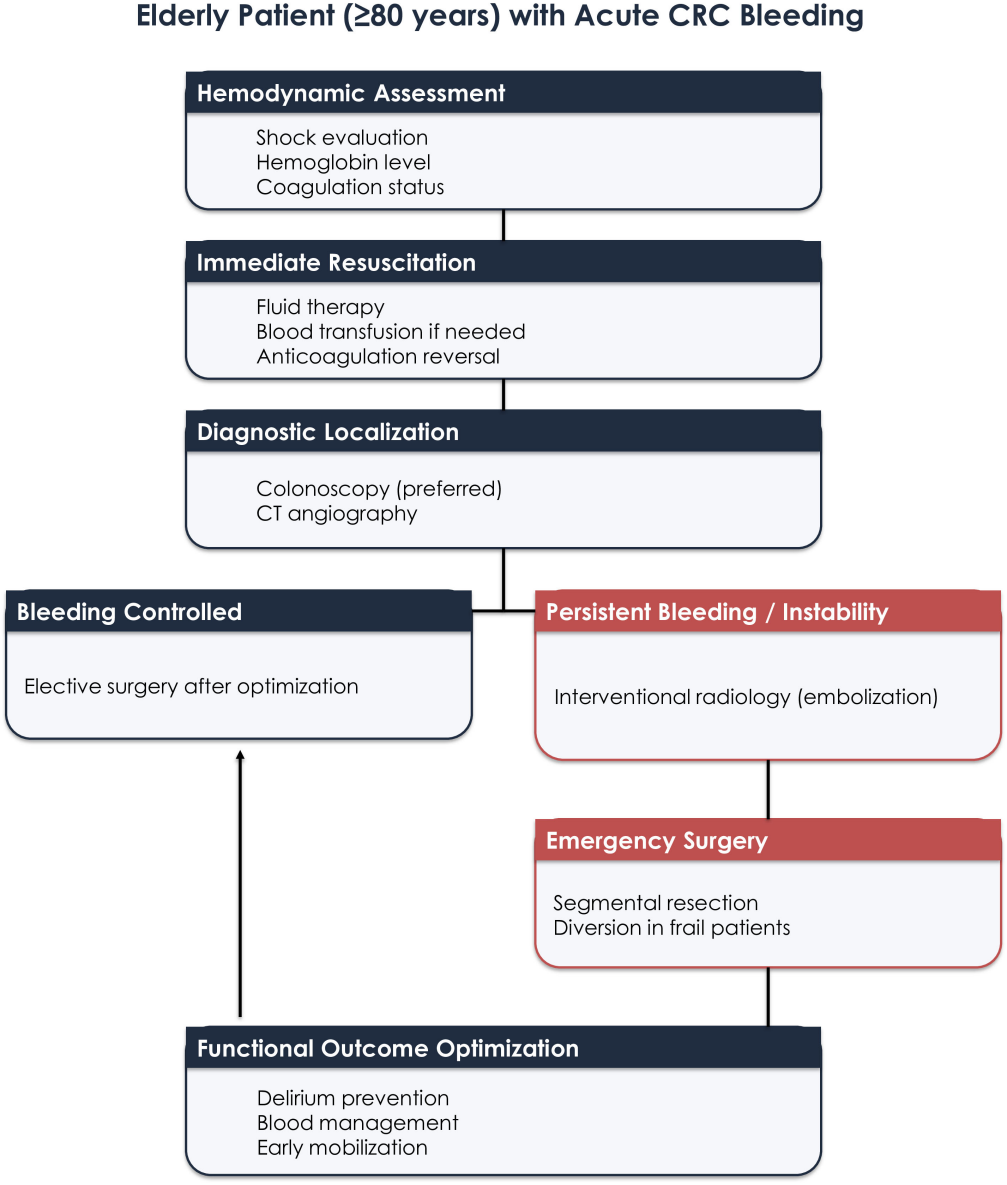
Management strategy for acute hemorrhage caused by colorectal cancer in elderly patients. The algorithm illustrates stepwise escalation from resuscitation and endoscopic hemostasis to interventional radiology and emergency surgery, incorporating functional outcome considerations.

Acute CRC-related bleeding is less frequent but may cause rapid deterioration, particularly in elderly patients with cardiovascular disease or anticoagulation therapy (20, 27) (**Figure 3**).

Presentation ranges from overt hemorrhage with instability to progressive anemia. Management includes prompt resuscitation, correction of coagulopathy, and individualized handling of anti-thrombotic agents (28).

Urgent endoscopy remains first-line for diagnosis and hemostasis, allowing potential conversion to elective surgery (29). If unsuccessful, selective arterial embolization offers an effective minimally invasive alternative (30, 31).

Emergency surgery should be reserved for uncontrolled bleeding and should prioritize stabilization rather than extensive oncologic resection. Delirium prevention, restrictive transfusion strategies, and early mobilization are central to preserving functional outcomes (32).

## Discussion

Emergency CRC diagnosis demonstrates a strong age-related gradient. Analyses from the International Cancer Benchmarking Partnership (ICBP) revealed that emergency presentation accounts for approximately 12% of rectal cancers, with significantly higher rates observed in patients aged ≥85 years (2).

Large registry-based studies further confirm these findings. In a SEER-Medicare cohort including more than 31,000 patients aged ≥80 years with colon cancer, nearly 46% were diagnosed during emergency hospitalization. Emergency presentation was associated with a marked reduction in survival, with one-year survival decreasing from 86% following elective diagnosis to approximately 70% after emergency presentation (3).

Emergency presentation negatively affects multiple aspects of oncologic management. These patients often undergo incomplete staging, are less likely to receive neoadjuvant or adjuvant therapies, and experience higher postoperative complication rates and longer hospitalization (33). Furthermore, emergency surgery is independently associated with increased mortality and reduced long-term cancer-specific survival.

### Determinants of clinical decision-making in the very elderly

Therapeutic decision-making in colorectal cancer emergencies among very elderly patients requires integration of tumor stage, acute clinical severity, comorbidity burden, and biological age.

Chronological age alone is insufficient to predict surgical tolerance. The Comprehensive Geriatric Assessment (CGA) represents a cornerstone for individualized risk stratification, integrating functional independence, cognition, nutrition, social support, and polypharmacy evaluation (8). Incorporating CGA into emergency settings facilitates multidisciplinary discussion and helps distinguish patients suitable for curative-intent surgery from those in whom proportional, damage-control, or palliative strategies are more appropriate.

In this context, redefining surgical intent when necessary does not represent therapeutic nihilism but rather alignment of operative strategy with realistic life expectancy and patient-defined priorities (35).

### Frailty and physiological reserve

Frailty is one of the strongest predictors of postoperative mortality, complications, prolonged hospitalization, and institutionalization in elderly patients, often exceeding chronological age in prognostic relevance. It reflects cumulative decline across multiple organ systems, resulting in diminished resilience to surgical and inflammatory stress.

Validated frailty indices incorporating functional performance, sarcopenia, nutritional status, cognition, and mobility provide more accurate outcome prediction and guide treatment intensity (33). Simple bedside tools such as gait speed, grip strength, and activities-of-daily-living assessment can rapidly identify vulnerable individuals who may benefit from tailored perioperative strategies or less invasive approaches.

Early recognition of frailty allows anticipation of predictable complications, including delirium, respiratory failure, and functional decline, enabling proactive geriatric co-management and early mobilization protocols (34).

In emergency colorectal surgery, frailty assessment supports a shift from procedure-centered radicality toward outcome-oriented proportionality, balancing oncologic benefit with realistic recovery potential.

### Comorbidity burden

Meta-analyses consistently demonstrate that comorbidities significantly increase early postoperative mortality and complication rates in elderly CRC patients. Odds ratios have been reported to reach 1.71 for patients with mild-to-moderate disease and up to 2.62 for individuals presenting with severe comorbidity burden (36).

Cardiovascular disease, including coronary artery disease and heart failure, represents one of the most impactful predictors of adverse surgical outcomes due to increased perioperative ischemic risk and hemodynamic instability. Chronic pulmonary disease contributes to elevated risk of postoperative respiratory failure and pneumonia, particularly after abdominal surgery. Renal insufficiency complicates fluid management and drug metabolism, while diabetes mellitus is associated with impaired wound healing and increased infection susceptibility. The presence of multiple concurrent diseases often results in polypharmacy, which may further complicate perioperative management through drug interactions and altered pharmacodynamics. Therefore, comprehensive preoperative optimization of comorbid conditions is essential to improve surgical tolerance and reduce complication rates (32).

Despite growing evidence, major knowledge gaps remain. Randomized trials specifically targeting patients aged ≥80 years are absent. Geriatric endpoints such as functional recovery, cognitive outcomes, and independence preservation are rarely incorporated into oncologic surgical trials (19, 32).

Addressing these gaps through predictive frailty-based triage models and prospective geriatric-oncology collaborative trials, integrating patient-reported outcomes and real-world registry data, is essential to align surgical decision-making with realistic oncologic benefit and patient-centered outcomes. Future research should prioritize prospective studies specifically designed for very elderly populations, integrating frailty metrics, functional outcomes, and patient-reported measures.

### Tumor stage and metastatic disease

The biological behavior and stage of CRC strongly influence treatment strategy selection. Advanced local disease or the presence of distant metastases frequently shifts treatment goals away from curative resection toward palliation and symptom control. In elderly patients, particularly those with limited physiological reserve, aggressive oncologic surgery may not provide meaningful survival benefit and may instead result in prolonged postoperative disability. Multidisciplinary evaluation incorporating radiologic staging, oncologic prognostic models, and geriatric assessment allows clinicians to balance survival expectations with potential treatment-related morbidity. In selected cases, non-operative management, endoscopic interventions, or systemic therapy may offer adequate symptom relief while minimizing procedural risk (37).

### Limitations

This review has several limitations. As a narrative review, it is subject to potential selection bias and does not provide quantitative synthesis of outcomes. Evidence specifically addressing patients aged ≥80 years remains limited, and many recommendations are extrapolated from younger populations. In addition, heterogeneity in frailty assessment tools and outcome reporting limits comparability across studies (38).

## Conclusions

Emergency colorectal cancer in patients aged ≥80 years represents a high-risk and increasingly prevalent clinical scenario requiring integration of oncologic rigor with geriatric-oriented care.

Bridge-to-surgery strategies improve outcomes in obstruction, while damage-control surgery remains essential in septic presentations. Frailty-informed proportionality and goal-oriented decision-making are central to optimizing functional recovery and quality of life.

Future research should prioritize geriatric-specific endpoints, predictive frailty-based triage models, and real-world functional outcomes to better align emergency surgical strategies with patient-centered goals.
